# Large Displacement Detection Using Improved Lucas–Kanade Optical Flow

**DOI:** 10.3390/s23063152

**Published:** 2023-03-15

**Authors:** Saleh Al-Qudah, Mijia Yang

**Affiliations:** Department of Civil, Construction and Environmental Engineering, North Dakota State University, Fargo, ND 58108, USA

**Keywords:** Lucas–Kanade optical flow, computer vision, displacement monitoring, convergence, template matching

## Abstract

Displacement is critical when it comes to the evaluation of civil structures. Large displacement can be dangerous. There are many methods that can be used to monitor structural displacements, but every method has its benefits and limitations. Lucas–Kanade (LK) optical flow is recognized as a superior computer vision displacement tracking method, but it only applies to small displacement monitoring. An upgraded LK optical flow method is developed in this study and used to detect large displacement motions. One motion controlled by a multiple purpose testing system (MTS) and a free-falling experiment were designed to verify the developed method. The results provided by the upgraded LK optical flow method showed 97 percent accuracy when compared with the movement of the MTS piston. In order to capture the free-falling large displacement, the pyramid and warp optical flow methods are included in the upgraded LK optical flow method and compared with the results of template matching. The warping algorithm with the second derivative Sobel operator provides accurate displacements with 96% average accuracy.

## 1. Introduction

Buildings, bridges, and dams are examples of complex structures that contribute to a society’s economic growth and quality of life. As these structures age and deteriorate, proper inspection, monitoring, and maintenance have become increasingly important for both safety and economic reasons. The traditional method of periodic human visual inspection is insufficient. Nondestructive evaluation (NDE) has demonstrated potential for detecting hidden damages, but the large size and number of structures make such a local inspection method difficult to implement.

A significant amount of research has been conducted in the field of structural health monitoring over the last two decades, with the goal of objective and quantitative structural damage detection and integrity assessment based on measurements by different sensors such as accelerometers [1,2,3,4]. Others expanded the method and extracted mode shapes through a power spectrum to identify the damage [5,6]. Although these research findings have enriched structural health monitoring (SHM) methods, their wide deployment in realistic engineering structures is restricted by the demand of complicated and costly sensor networks and data acquisition system establishment and preservation. Conventional contact-type displacement sensors such as linear variable differential transducers (LVDTs) require a stationary reference point, which is often difficult to find in the field.

To overcome these drawbacks, researchers have been actively exploring different sensor systems, such as wireless sensors or fiber optic sensors [7], that can promote the status of SHM [8]. Camera and computer vision-based sensors have demonstrated great potential for non-contact remote evaluation of structural responses in recent years, with displacements obtained by tracking the motions of targets in images. The system involves a camera for video capturing and a computer for data processing and analysis [9]. Both lab and field studies have verified the success of computer vision-based SHM methods such as using the motion of cables to determine cable tension forces [10]. 

With developments in computer vision in interdisciplinary fields, more and more efficient algorithms for structural displacement tracking are being proposed. Template matching using Zero-mean Normalized Cross Correlation (ZNCC), optical flow, and feature point matching are instances of these algorithms [11,12,13]. The Kanade–Lucas–Tomasi (KLT) optical flow method, which is recognized as a sparse optical flow method with good accuracy, has been successfully adopted in laboratory and field experiments [14,15]. The accuracy of KLT is reliant on the quality of feature locations. Corner detectors are typically used to identify these feature points, which include the Harris corner [16], Shi–Tomasi corner, scale-invariant feature [17], and speed-up robust feature detectors [18]. Zhu et al. [19] used the pyramid Lucas and Kanade (LK) optical flow to detect the displacements. No markers were used. However, the tracking was used for small displacements. It needs more research to use the LK optical flow method for monitoring large structural displacements.

This paper presents an innovative computer vision method for structural dynamic displacement tracking, based on the pyramid and warping LK optical flow. The Gaussian filter is first applied in image smoothing, which denoises the frame images and preserves the image features. The pyramid and warping LK optical flow is then used to obtain dynamic displacements even for fast and large movements. The developed method is verified through laboratory tests using the multipurpose test system (MTS) and 97% average accuracy is reached. A free-falling aggregate test is also performed to validate the effectiveness of the LK optical flow method on multiple large displacements, in which the moving objects are first segmented and then tracked. Moreover, the developed LK optical flow method is compared with the traditional feature tracking method, in which the center of gravity location of each segment was tracked in each image. Consistent results with higher accuracy are obtained for the LK optical flow method with the Harris corner detector compared to the traditional feature tracking method.

The content of this paper is presented as follows: Section 2 introduces the methodology and system development including image smoothing, target point choosing, target tracking, and displacement calculation. Experimental validation is presented in Section 3, and conclusions based on the results from the conducted tests are shown in Section 4.

## 2. Methods

### 2.1. General Description

**Figure 1:**
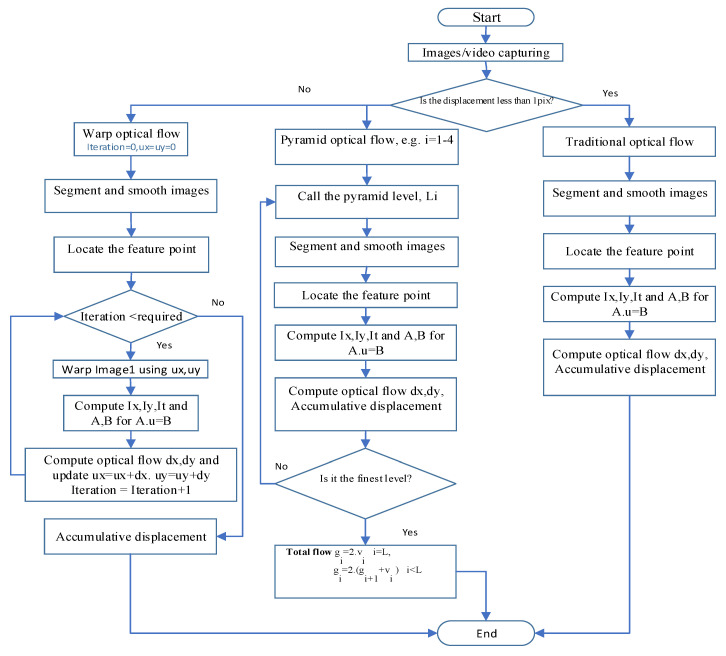
Flowchart of LK optical flow.

The hardware for the proposed vision-based system consists of a camera to capture images and video acquisition with specifications as shown in Table 1 and a computer for image analysis and displacement calculation. The software component includes the algorithms for image tracking and displacement calculations. Most of the code is written in MATLAB, R2020b and the ImageJ 1.54c program is sometimes used for verification purposes. The main steps of the LK optical method can be described as shown in Figure 1. First, the target video is captured and converted to images. Then, according to the possible displacement magnitude, one algorithm of the optical flow method can be chosen. After that, the images are smoothed, as described in Equation (1), using a low-pass filter to eliminate noises generated due to light intensity, shade, or shake of the camera. The feature points are then identified. In this paper, two types of feature points are used, which are the Harris corner in the first test and the centroid of the moving object ’aggregate’ in the second test. The optical flow algorithm is then used to evaluate the displacements of these points.

(1) $$f^{\prime }\left(x,y\right)=\sum_{i=-a}^{a}\sum_{i=-b}^{b}f\left(x+i,y+j\right)*h\left(i,j\right)$$

where *f*(*x*,*y*), *h*(*i*,*j*) and *f*′(*x*,*y*) are the image, the filter and the filtered image, respectively. 

### 2.2. Camera Calibration and World Coordinate

Distortion is affecting the accuracy of the calculations. Camera calibration is performed as a preliminary step to obtaining accurate coordinates or correcting distortion. For example, a traditional calibration method adopts a planar checkboard [20]. To implement this approach, it is necessary to take a variety of photos of the checkboard using the to-be-calibrated camera. The pinhole model is then used to establish the transformation from the real word to the image plane through Equation (2):

(2) $$\tilde{m}=A\left[R\;\;\;t\right]\tilde{M}$$

where $\tilde{m}$ = [*u*,*v*,1]^T^ is the image coordinates, *A*_3×3_ represents the camera intrinsic matrix, [*R t*] represents the camera extrinsic matrix, and $\tilde{M}$ = [*X*,*Y*,*Z*,1]^T^ is the world coordinates.

However, for cameras with no apparent lens distortion, the calibration step is not necessary. In general, for displacement monitoring, it is preferable to locate the target region in the central area of the field of view, which suffers less lens distortion [20]. So, in our experiments, the target was maintained in the center area of the field of view.

The actual displacement in real world compared to the displacement obtained in images is obtained through the scale factor, which can be calculated by Equation (3):

(3) $$SF=\frac{Z}{f}=\frac{D}{d}$$

where *Z* is the distance between the camera and the target object, *f* is the focal length of the lens, *D* and *d* denote the real and pixel length of the object, respectively.

### 2.3. Image Smoothing

Image noise is a random variable of pixels’ brightness in images due to the camera sensor, ISO settings, and shutter speed [21]. In fact, image noise is unavoidable with most cameras, and it is the primary technical issue when one wants to accurately estimate the optical flow field [22]. Image smoothing was applied through the convolution integral of the image with a Gaussian filter to reduce the noise. 

### 2.4. Image Derivative

Derivative or sharpening of image highlights transitions in image intensity. The strength of the response of a derivative operator is proportional to the magnitude of the intensity discontinuity at the point at which the operator is applied. Therefore, image derivative enhances edges and other discontinuities and de-emphasizes areas with slowly waring intensities. The first and second-order derivatives of two-dimensional function *f*(*x*,*y*) are the pixel intensity difference over two successive pixels as described in Equation (4) and Equation (5), respectively.

(4) $$\frac{df}{dx}=f\left(x+1,y\right)-f\left(x,y\right)$$

(5) $$\frac{d^{2}f\;}{dx^{2}}=f\left(x+1,y\right)+f\left(x-1,y\right)-2*f\left(x,y\right)$$

where *f*(.) is a function, and (*x* + 1), (*x*), and (*x* − 1) represent successive independent variables.

### 2.5. Feature Points Selection

In this paper, two types of feature points are selected which are the Harris corner and the centroid of targets. The Harris corner is independent of rotations and is regarded as the best corner detection method [16]. This corner can be found when there are two dominant and different edge directions in the local region of a point. Equations (6) and (7) can be used to find these locations.

(6) R=det(M)−k(trace(M))

(7) $$M=\left[\begin{array}{cc} I_{x}{}^{2} & I_{x}I_{y}\\ I_{x}I_{y} & I_{y}{}^{2} \end{array}\right]$$

where *k* = 0.04, as recommended by Harris (Harris and Stephens 1988). *I*_x_ and *I_y_* are image derivatives with respect to *x* and *y*, respectively. So, corners are the pixels that have *R* values greater than a specified threshold. The second type of feature, which is the centroid used in this paper, can be defined after segmentation of the object from the background.

### 2.6. Target Tracking

Target tracking is a process used in computer vision to recognize and track specific objects. There are many methods used for target tracking such as optical flow [14], template matching [23], and feature point matching [24]. However, template matching is not resistant to illusions such as light and shade, and it cannot be used to accurately track slender objects [20]. Feature point matching depends on sharp points by feature detector or descriptor. Optical flow estimation identifies motions or flows in all pixels within the target region, rather than searching for matching locations in a large region or a few key points. In this paper, modified LK optical flow is used and verified. Moreover, the template matching method is used as the third-party verification to the optical flow method. 

Unlike template matching, optical flow provides a template-free method. However, traditional optical flow fails when the target displacement becomes large. More robust methods in displacement monitoring are needed for cases such as tracking the fragmentation of a blasting event. Thus, an upgraded LK optical flow method is proposed in this paper through the addition of the second derivatives of images and the adoption of the Sobel operator. This operator applies at 0.5 variance, which makes the image intensity value at each location closer to the mean and yields more accurate results. 

#### 2.6.1. Optical Flow Method

The motion between two images is represented by optical flow, which is the velocity estimation of a video or image sequence. To determine the optical flow between two images, two fundamental assumptions are made, which are brightness constancy and small motion between image sequences. Optical flow is obtained in this study through the LK method [14]. The LK method can compute velocity from spatial and temporal derivatives of image intensity or from filtered versions of the image using a low-pass filter as shown in Equation (8):

(8) I(x,t)=I(x+δx,t+δt)

where ***I*** (*x*, *t*) represents image intensity. From Taylor’s expansion of Equation (8) or from the assumption that the image intensity is conserved, Equation (9) can be written as:

(9) $$\nabla I\left(x,t\right).u+I_{t}\left(x,t\right)=0$$

where ∇***I***(*x*, *t*) is the partial derivative of ***I*** (*x*, *t*) with respect to the image dimension in two directions, ***I****_t_* (*x*, *t*) is the partial derivative of ***I*** (*x*, *t*) with respect to time, and *u* is the optical flow vector in two dimensions. Equation (8) has two unknown variables, so it cannot be solved without other conditions. The LK method assumes that pixels within a window have the same velocity and applies Equation (10), the least square method, to calculate the displacement vector [14].

(10) $$E\left(u\right)=\Sigma {\left[\nabla I\left(x,t\right).u+I_{t}\left(x,t\right)\right]}^{2}$$

The true 2D optical flow *u* will lead to the least square error that minimizes *E*(*u*), which can be found through Equation (11) as follows:

(11) $$\left[\begin{array}{cc} \Sigma I_{x}{}^{2} & \Sigma I_{x}I_{y}\\ \Sigma I_{x}I_{y} & \Sigma I_{y}{}^{2} \end{array}\right]\left[\begin{array}{c} u_{x}\\ u_{y} \end{array}\right]=\left[\begin{array}{c} \Sigma I_{x}I_{t}\\ \Sigma I_{x}I_{t} \end{array}\right]$$

where all sums are taken over points in the neighborhood.

#### 2.6.2. Pyramid Optical Flow for Large Displacement Tracking

Pyramid optical flow is an innovation optical flow can be used to detect large motion by decreasing the size of the image. Pyramid optical flow method is similar to the traditional optical flow, except it reduces the resolution of the image three or four times. Then, the optical flow between images at the same level is calculated using the traditional optical flow and the flow between levels is calculated using either Equation (12) or Equation (13):

(12) $$g_{i}=\left(2v_{i\;}\right)\;,\;i=L$$

(13) $$g_{i}=2\left(v_{i\;}+g_{i-1}\right)\;,\;i<L$$

where *L* is the finest level and *i* is the current level. ***v****_i_* is the optical flow from the current level and ***g****_i_*_−1_ is the optical flow from the previous level. As the resolution of the image is reduced, the displacement is reduced too. So, the pyramid optical flow method finds the optical flow at each level and combine them together to detect larger displacements.

#### 2.6.3. Warp Optical Flow for Large Displacement Tracking

Warp optical flow method is a unique method that tries to detect large motions through the difference between successive images by warping. In this algorithm optical flow is calculated through an iteration process using the traditional optical flow, since the calculated flow is not convergent to the final flow yet so the flow at this step is used to shift the image to the new position. Then, optical flow will be calculated again through the adjusted images that were warped with the calculated flow. This process will continue until the final flow reaches a fixed value and the difference between the optical flows at two successive steps are minimized [19]. 

### 2.7. Template Matching Method

Template matching method is used in this paper to verify the LK optical flow method. The general concept behind the template matching is to slide a chosen area as a template across another image and find the best match by calculating the similarity between the template and the overlapped region of the image while sliding. There are two primary categories of template matching methods: sum of squared differences (*SSD*) and cross-correlation methods. Both of these categories are widely used in a variety of applications [25]. In this study, the sum of squared differences is applied using Equation (14):

(14) $$SSD=\Sigma {\left[T\left(x\prime ,y\prime \right)-I\left(x+x^{\prime },y+y\prime \right)\right]}^{2}$$

where ***T*** and ***I*** are the grayscale image intensity of template and overlapped images, respectively; (*x*, *y*) and (*x*′, *y*′) denote the location coordinates in the template and the overlapped image, respectively. SSD computes the sum of the squared difference between the intensities of the template and these of the overlapped image. The greatest similarity can be found when the difference is the smallest.

## 3. Experiments and Results

### 3.1. Method Verification

**Figure 2:**
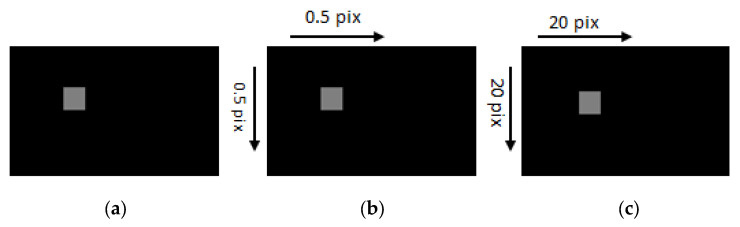
Patch test. (**a**) Original patch. (**b**) Patch shifted by 0.5 pix in both directions. (**c**) Patch shifted by 20 pix in both directions.

For validation, two tests were applied to verify each optical flow method, namely, traditional, pyramid, and iterative warping methods. Two patches were drawn, and translations were applied using MATLAB. One patch is moved 0.5 pixels in both directions, and the traditional optical flow was used to find the motion. The other patch is translated with 20 pixels in both directions, and the displacement is calculated using the pyramid and warp optical flow, these patches are shown in Figure 2. The displacement found through the three optical flow methods are shown in Table 2.

As we can see in Table 1, as long as there is a small displacement, traditional optical flow works well, but with a large displacement, traditional optical flow fails. Instead, the pyramid and warp optical flow work better.

### 3.2. Experimental Setup

Two tests were performed in this study to check the performance of the proposed methods in real applications. The first test verified the traditional optical flow method, while the second test tracked large movement displacements of free-falling aggregates. In the first test, a camera was used to record a video at 60 frames per second with 4 K resolution when the MTS machine was moving with an amplitude of 12.7 mm at a frequency of 0.5 Hz following a sine wave. A Gaussian filter was then used to decrease the noise and minimize aliasing. However, for the second test, a high-speed camera, which is shown in (Table 3), was used to record a video of free-falling aggregates.

### 3.3. Measurement and Analysis Results

**Figure 3:**
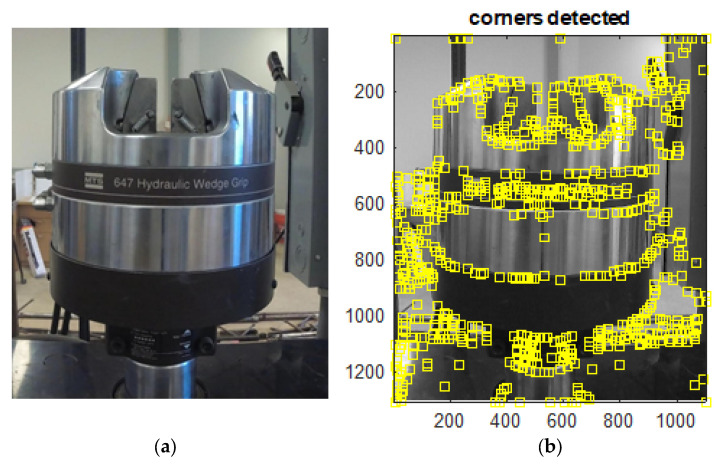
The moving piston of the MTS machine. (**a**) Before feature detection. (**b**) After feature detection.

**Figure 4:**
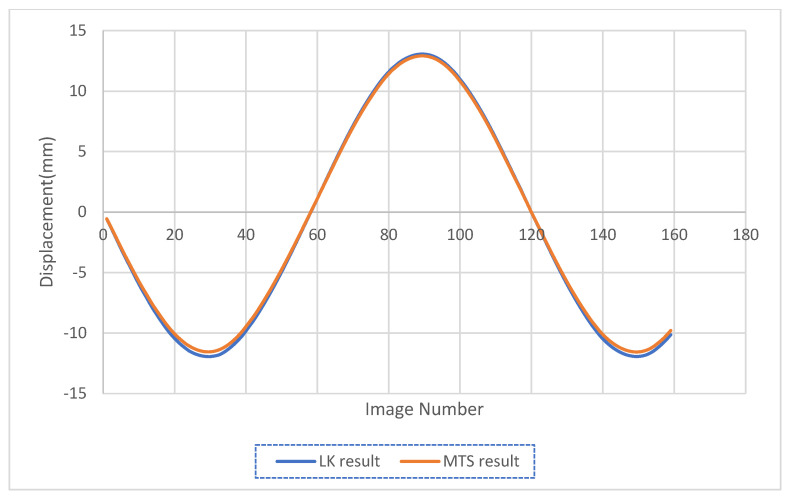
Displacement obtained using the direct LK optical flow, compared with the output of the MTS machine movement.

In the first test, Harris corners were found on the MTS and tracked by the traditional LK optical flow. Figure 3 shows the piston before and after corners detection. The vibration was induced at the moving piston. The time history results from the LK optical flow are shown in Figure 4, compared with the output of the MTS machine movements. The results of the LK method coincide with the MTS displacement most of the operation time. There are some small deviations at large monitored displacements with respect to the true movements.

**Figure 5:**
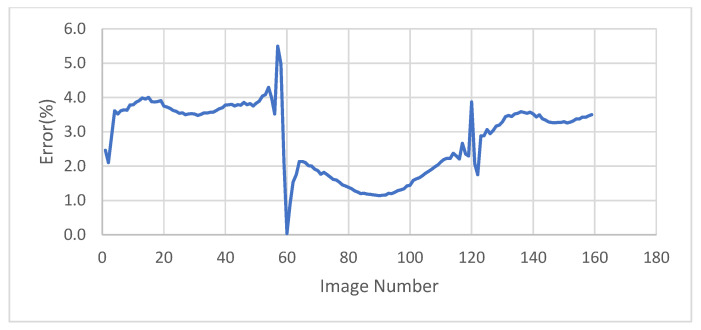
Error time history of the test on tracking the MTS machine movement.

The accuracy of the test was checked at points of interest, which are the peaks points and x-intersection points, and these results are shown in Table 4. Most of these results range from 95–98% in accuracy, while the average accuracy for all results in the first test is 97%. Error time history is shown in Figure 5.

**Figure 6:**
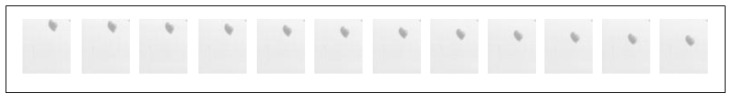
The first 12 successive images of the free-falling aggregate test.

**Figure 7:**
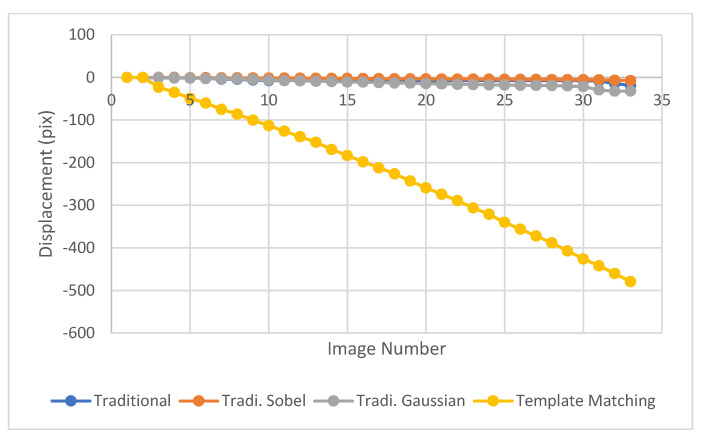
Displacement of a free-falling aggregate obtained using the 1st and 2nd derivative LK optical flow, compared with the displacement obtained using the template matching method.

In the second test, an aggregate was released at rest and the high-speed camera was used to capture the movement, the first 12 successive images are shown in Figure 6. The traditional LK optical flow was used to track the motion of a free-falling aggregate, with the centroid of the aggregate as the feature point, but it could not detect the accurate displacement due to large movements. Additionally, the second derivative of image intensity was obtained using the Gaussian and Sobel operators and used with the traditional LK optical flow method but they did not converge as can be seen in Figure 7.

**Figure 8:**
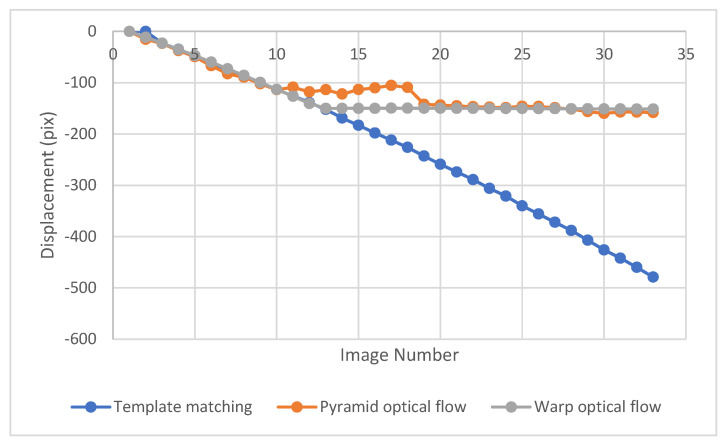
Displacement of a free-falling aggregate obtained using the pyramid and warp LK optical flow, compared with the displacement obtained using the template matching method.

The pyramid and warp LK optical flow methods were used to track the motion of a free-falling aggregate. The displacement results are shown in Figure 8 and compared with the template matching results. These results show that the pyramid LK optical flow tracks well until the 10th image, but it diverges from the correct path due to the large displacement after the 10th image; while the warp LK optical flow converges till the 12th image. So, both methods detect displacement to a certain level because they used image gradient to find the displacement.

**Figure 9:**
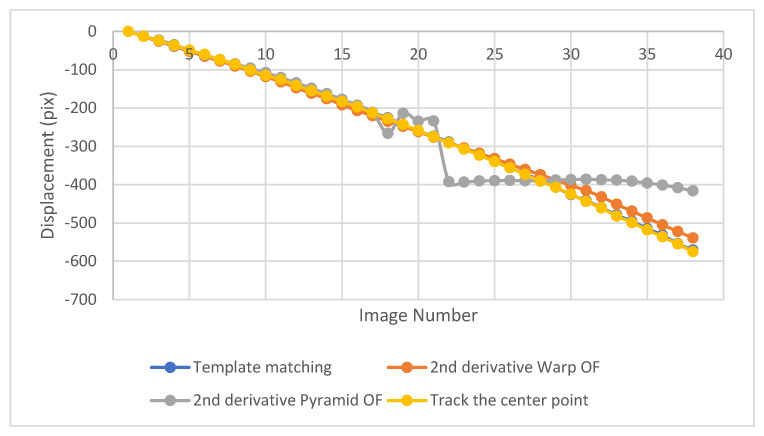
Displacement of a free-falling aggregate obtained using the pyramid and warp Sobel LK optical flow, compared with the displacement obtained using the template matching method.

In order to detect larger displacement for the second test, Sobel operators were applied to image gradients which obtained using Gaussian operators. The resultant displacement monitoring gave more accurate results especially after 14th image as can be seen in Figure 9. As Sobel operators using 0.5 variance where the image intensity values were closer to the mean and even closer to each other by applying this filter, warp optical flow method showed better convergences. However, that was not the case using the pyramid optical flow—it detects convergent results until the 17th image only, where pyramid Sobel optical flow could not catch the correct displacement after that which might be due to resolution decrement which reduce the accuracy with higher derivative. An additional tracking method is included for comparison, in which a MATLAB code was written to extract the location of the centroid of the target in each image and track it directly.

The accuracy for the second test, the free-falling aggregate test, using the warp optical flow has a 96% average value, but when the pyramid optical flow is used, the average accuracy is 96% till image number 17, then the average accuracy decreases to 89%. It is worthy to note the displacement derived through the center point method and/or the template matching was taken as the true displacement. Based on that, the accuracies of the pyramid and warp optical flow method are calculated.

## 4. Application of the Method in Monitoring a Solar Frame

**Figure 10:**
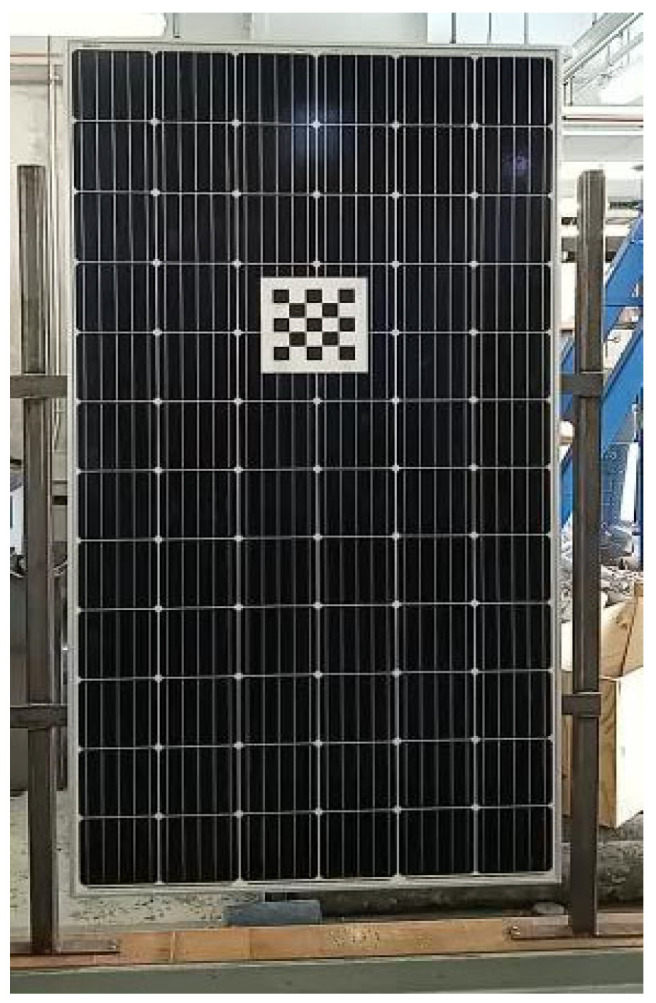
The solar frame used to monitor the true displacement induced by force.

**Figure 11:**
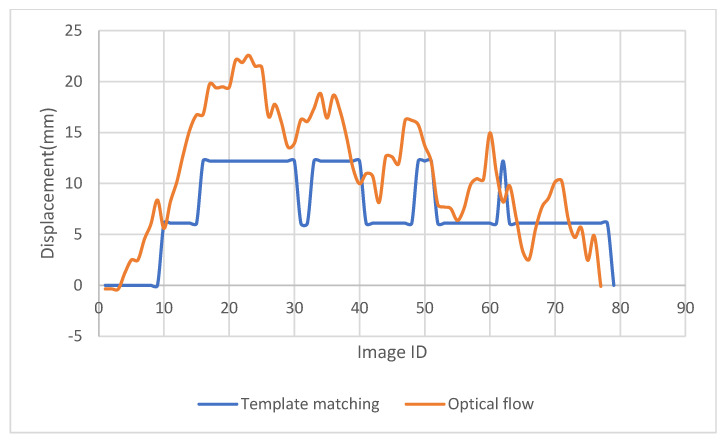
X-displacement of the solar frame detected using the modified LK optical flow method and template matching.

**Figure 12:**
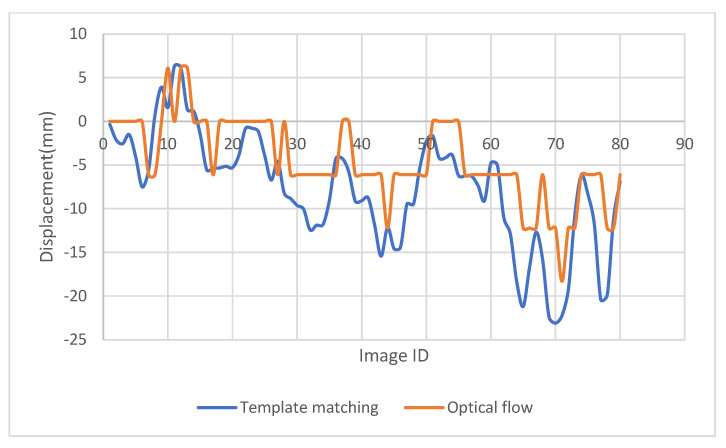
Y-displacement of the solar frame detected using the modified LK optical flow method and template matching.

To check the potential application of the proposed method, monitoring of a solar frame is conducted. The movement of the solar frame is excited through wind. A checkerboard with a size of 8.5 × 11 in. is used for the template matching purpose and was attached to one of the solar panels (Figure 10). A video of the solar frame was captured at 60 frames per minute, and the displacement of the solar frame was extracted through the images captured using the proposed LK optical flow and templated matching in both x, y directions (Figure 11 and Figure 12).

As can be seen in Figure 11 and Figure 12, the proposed optical flow gave more smooth and accurate results in displacement detection, compared with the displacement obtained through the template matching method. Template matching method cannot track the displacement accurately when the displacement increment is not an integer number of pixels, but provides a close estimation. The flat segments of Figure 11 and Figure 12 prove it. If the target is moving fast with a large displacement, both the traditional optical flow and the template matching method will face challenges, but the modified LK optical flow method could provide accurate results.

## 5. Conclusions

In this study, the pyramid and warp LK optical methods were proposed to track large displacements. The traditional LK optical flow was first applied to detect the dynamic displacement of a piston moving at 0.5 Hz within the MTS machine. The results show 97% accuracy among all images which were used to find the motion. The modified LK optical flow method was then used to detect the motion of a free-falling aggregate. The found displacements are compared with that of the template matching method. It was found that the modified LK optical flow method with Sobel operators can track large displacements, such as free-falling motions, with 96% average accuracy. A third test on a solar frame was used to confirm the effectiveness of the proposed method, where it can provide more accurate results when displacement increment is not an integer pixel number. 

For future work, as LK optical flow depends on Taylor series, more robust results can be found by taking additional terms of the Taylor expansion. The usage of different kind of operators could help as well. 

## Figures and Tables

**Table 1:** Specifications of the Yi 4k action camera used.

Main Processor	Ambarella A95E chipset, 32-MPixel image sensor pipeline, and an advanced encoder with 4k resolution
Image Sensor	Sony IMX377, ½._3′_′, 12 Megapixels CMOS image sensor
LCD Screen	2.19″, 640 × 360 resolution, 330 ppi, 250 cd/m^2^ brightness, 30 FPS, 160″ FOV, 16:9
Lens	F2.B aperture/155″ wide angle lens, 7G, f = 2.66 ± 5% mm
Lens distortion correction	100% of the wide angle lens distortion correction

**Table 2:** Results of validation tests of the optical flow algorithms.

Test Method	True Displacement		Traditional OF		Pyramid OF		Warp OF	
Direction	x	y	x	y	x	y	x	y
First test	0.5 pix	0.5 pix	0.5 pix	0.51 pix	---	---	---	---
Second test	20 pix	20 pix	2.6 pix	2.6 pix	22 pix	24 pix	20 pix	20 pix

**Table 3:** Specifications of the high-speed FASTEC IL5 camera used.

Sensor	12-Bit CMOS Sensor with 5 µm Square Pixels
Max resolution	1920 × 1080
Light sensitivity	1600 to 12,800″ iso monochrome, 800 to 6400″ iso color
Shutter	3 µs to 41.654 ms

**Table 4:** Accuracy of the traditional LK method with respect to the MTS movements.

POI	x-Inter. 1	Min pt. 1	x-Inter. 2	Max. pt.	x-Inter. 2	Min pt. 2
Accuracy	97.5	96.5	95.0	98.8	96.1	96.7

Note abbreviations are: POI: point of interest, x-inter: intersection with x axis, min pt.: minimum point in the curve, max pt.: maximum point in the curve and the numbers stands for the first and second interactions or minimum/maximum points.

## Data Availability

Some or all data, models or code that support the findings of this study are available from the corresponding author upon reasonable request.

## References

[1] Tian B., Liu H., Yang N., Zhao Y., Jiang Z. (2016). Design of a Piezoelectric Accelerometer with High Sensitivity and Low Transverse Effect. Sensors.

[2] Wang H., Li A., Guo T., Tao T. (2014). Establishment and Application of the Wind and Structural Health Monitoring System for the Runyang Yangtze River Bridge. Shock Vib..

[3] Wang H., Tao T., Guo T., Li J., Li A. (2014). Full-scale measurements and system identification on Sutong cable-stayed bridge during typhoon Fung-Wong. Sci. World J..

[4] Zhu L., Fu Y., Chow R., Spencer B.F., Park J.W., Mechitov K. (2018). Development of a High-Sensitivity Wireless Accelerometer for Structural Health Monitoring. Sensors.

[5] Qu C.X., Yi T.H., Li H.N., Chen B. (2018). Closely spaced modes identification through modified frequency domain decomposition. Measurement.

[6] Qu C.X., Yi T.H., Li H.N. (2019). Mode identification by eigensystem realization algorithm through virtual frequency response function. Struct. Control Health Monit..

[7] Li S., Wu Z. (2007). Development of distributed long-gage fiber optic sensing system for structural health monitoring. Struct. Health Monit..

[8] Li J., Mechitov K.A., Kim R.E., Spencer B.F. (2016). Efficient time synchronization for structural health monitoring using wireless smart sensor networks. Struct. Control Health Monit..

[9] Xu Y., Brownjohn J.M.W. (2018). Review of machine-vision based methodologies for displacement measurement in civil structures. J. Civil Struct. Health Monit..

[10] Kim S.W., Jeon B.G., Kim N.S., Park J.C. (2013). Vision-based monitoring system for evaluating cable tensile forces on a cable-stayed bridge. Struct. Health Monit..

[11] Dong C.Z., Celik O., Catbas F.N., O’Brien E.J., Taylor S. (2020). Structural displacement monitoring using deep learning-based full field optical flow methods. Struct. Infrastruct. Eng..

[12] Khuc T., Catbas F.N. (2017). Computer vision-based displacement and vibration monitoring without using physical target on structures. Struct. Infrastruct. Eng..

[13] O’Byrne M., Ghosh B., Schoefs F., O’Donnell D., Wright R., Pakrashi V. (2015). Acquisition and analysis of dynamic responses of a historic pedestrian bridge using video image processing. J. Phys. Conf. Ser..

[14] Lucas B.D., Kanade T. Iterative Image Registration Technique with an Application to Stereo Vision. Proceedings of the 7th International Joint Conference on Artificial Intelligence.

[15] Lydon D., Lydon M., del Rincon J.M., Taylor S.E., Robinson D., O’Brien E., Catbas F.N. (2018). Development and field testing of a time-synchronized system for multi-point displacement calculation using low-cost wireless vision-based sensors. IEEE Sens. J..

[16] Harris C., Stephens M. A combined edge and corner detector. Proceedings of the 4th Alvey Vision Conference.

[17] Lowe D.G. (2004). Distinctive image features from scale-invariant keypoints. Int. J. Comput. Vis..

[18] Dong C.Z., Celik O., Catbas F.N. (2019). Marker-free monitoring of the grandstand structures and modal identification using computer vision methods. Struct. Health Monit..

[19] Zhu J., Lu Z., Zhang C. (2021). A marker-free method for structural dynamic displacement measurement based on optical flow. Struct. Infrastruct. Eng..

[20] Xu Y., Brownjohn J., Kong D. (2018). A non-contact vision-based system for multipoint displacement monitoring in a cable-stayed footbridge. Struct. Control Health Monit..

[21] Boncelet C. (2009). Image Noise Models. The Essential Guide to Image Processing.

[22] Aslani S., Mahdavi-nasab H. (2013). Optical Flow Based Moving Object Detection and Tracking for Traffic Surveillance. Int. J. Electr. Comput. Energetic Electron. Commun. Eng..

[23] Tanimoto S.L. (1981). Template matching in pyramids. Comput. Graph. Image Process..

[24] Shi G., Xu X., Dai Y. SIFT feature point matching based on improved RANSAC algorithm. Proceedings of the 2013 5th International Conference on Intelligent Human-Machine Systems and Cybernetics (IHMSC).

[25] Dong C.Z., Catbas F.N. (2021). A review of computer vision–based structural health monitoring at local and global levels. Struct. Health Monit..

